# Chalcone Attenuates *Staphylococcus aureus* Virulence by Targeting Sortase A and Alpha-Hemolysin

**DOI:** 10.3389/fmicb.2017.01715

**Published:** 2017-09-06

**Authors:** Bing Zhang, Zihao Teng, Xianhe Li, Gejin Lu, Xuming Deng, Xiaodi Niu, Jianfeng Wang

**Affiliations:** ^1^Key Laboratory of Zoonosis, Ministry of Education, College of Veterinary Medicine, Jilin University Changchun, China; ^2^Center of Infection and Immunity, The First Hospital, Jilin University Changchun, China

**Keywords:** *Staphylococcus aureus*, sortase A, alpha-hemolysin, chalcone, inhibitor

## Abstract

*Staphylococcus aureus* (*S*.aureus) resistance, considered a dilemma for the clinical treatment of this bacterial infection, is becoming increasingly intractable. Novel anti-virulence strategies will undoubtedly provide a path forward in combating these resistant bacterial infections. Sortase A (SrtA), an enzyme responsible for anchoring virulence-related surface proteins, and alpha-hemolysin (Hla), a pore-forming cytotoxin, have aroused great scientific interest, as they have been regarded as targets for promising agents against *S. aureus* infection. In this study, we discovered that chalcone, a natural small compound with little anti-*S. aureus* activity, could significantly inhibit SrtA activity with an IC_50_ of 53.15 μM and Hla hemolysis activity with an IC_50_ of 17.63 μM using a fluorescence resonance energy transfer (FRET) assay and a hemolysis assay, respectively. In addition, chalcone was proven to reduce protein A (SpA) display in intact bacteria, binding to fibronectin, formation of biofilm and *S. aureus* invasion. Chalcone could down-regulate the transcriptional levels of the *hla* gene and the *agrA* gene, thus leading to a reduction in the expression of Hla and significant protection against Hla-mediated A549 cell injury; more importantly, chalcone could also reduce mortality in infected mice. Additionally, molecular dynamics simulations and mutagenesis assays were used to identify the mechanism of chalcone against SrtA, which implied that the inhibitory activity lies in the bond between chalcone and SrtA residues Val168, Ile182, and Arg197. Taken together, the *in vivo* and *in vitro* experiments suggest that chalcone is a potential novel therapeutic compound for *S. aureus* infection via targeting SrtA and Hla.

## Introduction

*Staphylococcus aureus* (*S. aureus*) is the etiologic agent of a wide range of clinical infections, including bacteremia, infective endocarditis, and osteoarticular infections, as well as skin and soft tissue infections and metastatic abscess formation (Lowy, 1998; Coates et al., 2014). The continuous emergence of *S. aureus* strains developing a resistance to antibiotics, such as methicillin-resistant *S. aureus* (MRSA) and vancomycin-resistant *S. aureus* (VASA), has been largely responsible for the severe clinical complications and increase in the incidence rate of unfavorable prognoses (Ippolito et al., 2010; Gould, 2013). Simultaneously, there are few high-efficiency antibiotics in the drug discovery pipeline. Thus, treatment options are severely limited. The pressing challenge is the identification of new drug targets and the discovery of new agents against *S. aureus* infection.

A formidable array of secreted exotoxins and surface proteins anchored in the cell wall plays a crucial role in the pathogenic process of *S. aureus* (Dinges et al., 2000; Wardenburg et al., 2007). Among all of the virulence factors, sortase and alpha-hemolysin (Hla) generate the most interest. Interest in sortase as a target for the establishment of anti-virulence strategies primarily stems from studies in which loss of the gene encoding sortase led to the decreased virulence of *S. aureus* in a mouse model of *S. aureus* infection (Albus et al., 1991; Mazmanian et al., 2000). As the so-called “house-keeping” sortase, sortase A (SrtA) plays a critical role in the anchoring of surface proteins of *S. aureus* to the cell wall envelope. Surface proteins of Gram-positive bacteria, as one of the virulence factors, play a significant part in the process of invading the host. One of the shared features of these proteins that mediate bacterial adhesion and evade host immune defenses is that they all contain LPXTG (Leu-Pro-X-Thr-Gly) sorting signals, which SrtA can identify and use to catalyze the anchor further (Fischetti et al., 1990; Scott and Barnett, 2006). Soon after SrtA was discovered and cloned, many studies reported that the *in vivo* inhibition of SrtA could be measured. Subsequently, a number of SrtA inhibitors, including natural products and synthetic small molecules, among others, were identified (Clancy et al., 2010; Zhang et al., 2014, 2016; Lin et al., 2015).

Another important target, Hla, which is encoded by the *hla* gene and is generally secreted late in the exponential phase of growth, is a water-soluble pore-forming cytotoxin leading to the damage and death of cells, such as erythrocytes and epithelial cells, owing to its lytic property (Berube and Bubeck, 2013). Previous studies reported that Hla damaged the air-blood barrier of the lung in a rat model, and similar to sortase, *S. aureus* lacking Hla exhibited an obvious attenuated pathogenicity in a mouse model of *S. aureus* infection (Mcelroy et al., 1999; Wardenburg et al., 2007). A number of previous studies have shown that inhibitors targeting Hla could significantly prevent MASA infection, indicating a novel and effective strategy for combating *S. aureus* (Ragle et al., 2010).

**Figure 1:**
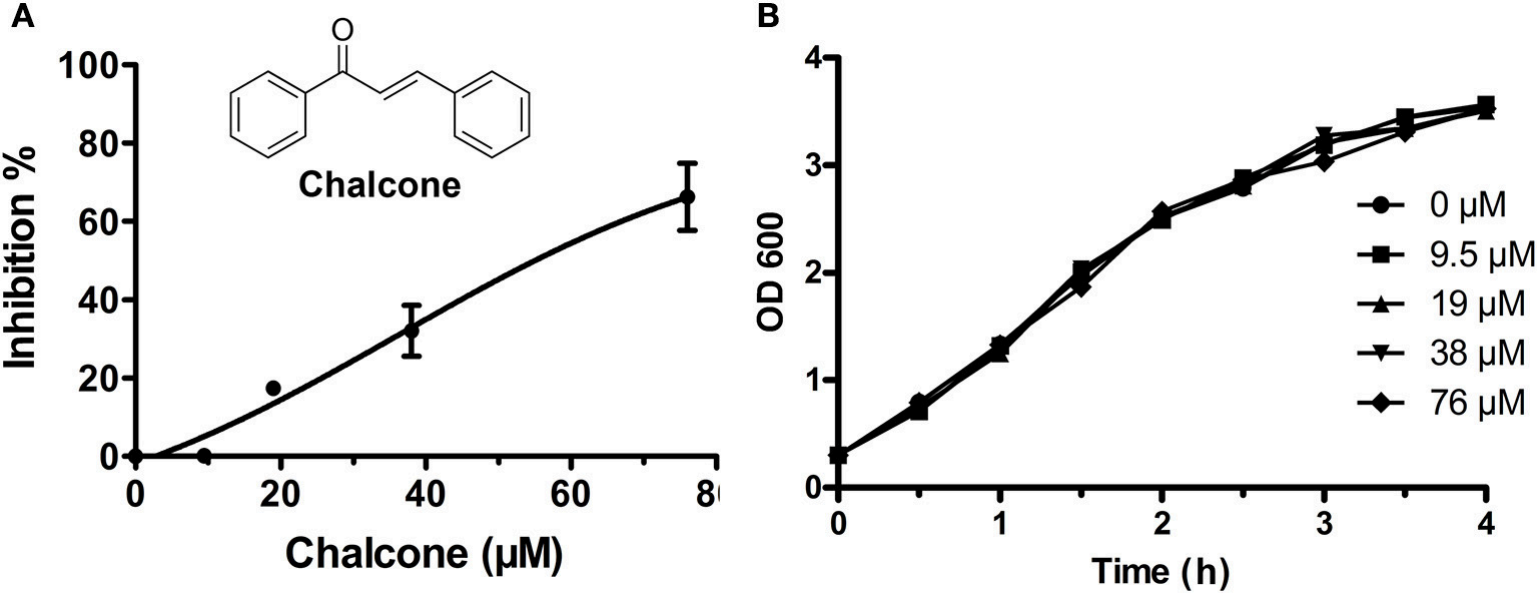
Chalcone inhibits *S. aureus* SrtA activity. **(A)** The inhibitory effect of chalcone on *S. aureus* SrtA activity. After pre-incubation with various concentrations of chalcone, followed by adding the model substrate peptide Dabcyl-QALPETGEE-Edans, a microplate reader was used to determine the catalytic activity of each sample. **(B)** The growth curve of *S. aureus* treated with the indicated concentrations of chalcone.

Notably, an anti-virulence strategy targeting sortase or Hla was able to disrupt the pathogenesis of bacterial infections without a direct interference with bacterial growth (Levy et al., 1976) and, thus, may be less likely to induce selective pressures and may slow down the development of drug resistance. In addition, we reasoned that a strategy simultaneously targeting both sortase and Hla could cause a double blow to *S. aureus* infection and thereby represent a more effective anti-infection treatment. In this study, we first found that one class of dietary compound, called chalcone (Figure 1A), could be a promising inhibitor for targeting both *S. aureus* SrtA and Hla and then systematically evaluated the inhibitory activity of chalcone with a fluorescence resonance energy transfer (FRET) assay and a hemolysis assay, respectively, where chalcone was found to significantly neutralize SrtA activity and inhibit Hla production. The therapeutic effect in a *S. aureus* infection mouse model was found to be obvious as well. With these approaches, we provide powerful evidence that chalcone is a potential agent for the treatment of *S. aureus* infection via targeting SrtA and Hla.

## Methods

### Bacterial strains, growth conditions, and reagents

*Staphylococcus aureus* strain USA 300 and *S. aureus* ΔSrtA strain USA 300 ΔSrtA, obtained from our lab, were used in the present study and cultured in brain-heart infusion (BHI) broth (Sigma) at 37°C. Chalcone was purchased from the Tianjin Yifang S&T Co., Ltd. (Tianjin, China). The fluorescent peptide Dabcyl-QALPETGEE-Edans was purchased from GL Biochem (Shanghai, China).

### Construction of plasmids encoding wild-type (WT)-SrtA, V168A-SrtA, I182A-SrtA, and R197A-SrtA

The DNA sequence encoding the WT-SrtA protein was amplified from *S. aureus* USA 300 genomic DNA and used as a template along with the corresponding primers (Table 1) to amplify the *S. aureus* SrtA sequence through PCR. The PCR products were digested with Nde1 and BamH1 and then cloned into the pGEX-6P-1 expression vector. The pGEX-6P-1-*srtA* plasmid encoding WT-SrtA was generated after the sequence was confirmed by DNA sequencing. Site-directed mutagenesis for V168A-SrtA, I182A-SrtA, and R197A-SrtA was carried out using the QuickChange site-directed mutagenesis kit (Stratagene, La Jolla, CA, USA). The mutagenic primer pairs employed to produce the three mutants are listed in Table 1.

**Table 1:** Oligonucleotide primers used in this study.

**Primer name**	**Oligonucleotide(5–3)^a^**
WT-SrtA-F	GCGGGATCCCAAGCTAAACCTCAAATTCC
WT-SrtA-R	CCGCTCGAGTTATTTGACTTCTGTAGCTACAA
V168A-SrtA-F	CCTACAGATGTAGGAGCGCTAGATGAACAAAAAGG
V168A-SrtA-R	CCTTTTTGTTCATCTAGCGCTCCTACATCTGTAGG
I182A-SrtA-F	GATAAACAATTAACATTAGCGACTTGTGATGATTACAATG
I182A-SrtA-R	CATTGTAATCATCACAAGTCGCTAATGTTAATTGTTTATC
R197A-SrtA-F	GACAGGCGTTTGGGAAAAAGCGAAAATCTTTGTAGCTACAG
R197A-SrtA-R	CTGTAGCTACAAAGATTTTCGCTTTTTCCCAAACGCCTGTC

a *Restriction endonuclease recognition sites or mutated codons are underlined*.

### Expression and purification of WT-SrtA, V168A-SrtA, I182A-SrtA, and R197A-SrtA

WT-SrtA and all mutant constructs were transformed into *Escherichia coli* BL21 (DE3) cells. Subsequently, the transformants were grown and selected in Luria-Bertani (LB) media with ampicillin (100 mg/L) at 37°C. To induce protein expression, 1 mM IPTG (Sigma) was added into the bacteria suspension when the OD (600 nm) reached 0.6–0.8, followed by overnight growth at 16°C. The bacteria were harvested through centrifugation at 3,000 × g for 30 min at 4°C and resuspended in the reaction buffer (50 mM Tris-HCl, 5 mM CaCl_2_ and 150 mM NaCl, pH 7.5). The cell debris was removed through centrifugation at 10,000 × g for 1 h at 4°C after sonication. The supernatant was applied to a self-packaged GST-affinity column (2 ml glutathione Sepharose 4B) (GE Amersham Biosciences, Piscataway, NJ, USA). First, the reaction buffer was used to remove the unbound contaminating proteins, then Precision Protease was used to digest the GST-tagged protein at 4°C overnight. Finally, the reaction buffer was used again to wash off the target protein. The point mutations V168A-SrtA, I182A-SrtA, and R197A-SrtA were expressed and purified similarly to WT-SrtA.

### WT and mutant SrtA activity measurement

To measure the activity of WT and mutant SrtA, a FRET assay was used. As detailed earlier (Tonthat et al., 1999), 100 μl of a mixture consisting of the reaction buffer, purified proteins and different concentrations of chalcone were added in the appropriate order to 96-well plates and incubated for 30 min at 37°C. Subsequently, upon the addition of the fluorescent peptide substrate Dabcyl-QALPETGEE-Edans, the reaction was initiated, and after incubation for 1 h at 37°C, the fluorescence was read using emission and excitation wavelengths of 350 and 520 nm, respectively.

### *Anti-S. aureus* activity of chalcone

The minimum inhibitory concentration (MIC) of chalcone against *S. aureus* was determined by broth microdilution according to the NCCLS guideline M_31_-A_2_. For growth curve plotting, 1 ml of the overnight bacterial cultures was transferred (1: 50) to BHI broth containing different concentrations of chalcone. The absorbance reading was taken at OD (600 nm)_._

### Protein A (SpA)-related fluorescence analysis

Overnight cultures of *S. aureus* wild-type strain (WT strain) were inoculated 1: 1000 into fresh BHI broth and grown to an OD (600 nm) of 1.0 with chalcone or dimethyl sulfoxide (DMSO, as the solvent control) at 37°C. Meanwhile, the USA300 ΔSrtA strain (WTΔSrtA strain) was used as the positive control. The bacteria were fixed with a 4% formaldehyde solution for 20 min after centrifugal collection (3,000 × g for 5 min) and washed twice with PBS. The bacteria were then resuspended in PBS containing a 1: 25 dilution of FITC-labeled goat anti-rabbit IgG (eBioscience) and incubated for 2.5 h at room temperature. Subsequently, the cells were washed three times with PBS and added to poly-L-lysine-coated glass slides. The SpA-related fluorescence was observed using a confocal laser-scanning microscope (Olympus, Shanghai, China).

### Fibronectin-binding assay

The WT strain was grown in BHI broth to an OD (600 nm) of 0.5 with chalcone or DMSO in a shaking incubator with a shaker rate of 190 rpm at 37°C; the WTΔSrtA strain was used as a positive control. The bacteria were collected centrifugally (3,000 × g for 5 min), and after washing with PBS three times, the cells were resuspended using PBS and adjusted to an OD (600 nm) of 1.0. Fibronectin from bovine plasma with a concentration of 2 μg/ml was added into 96-well flat-bottom polystyrene microtiter plates, 100 μl in every well, and incubated overnight at 4°C. After washing three times with PBS, 100 μl of bovine serum albumin (BSA) at a concentration of 5% was added into the plate and incubated for 2 h at 37°C. The plates were washed three times afterwards, then 100 μl of the bacterial suspension was added and incubated for 2 h at 37°C. Subsequently, after removing the suspension and washing three times with PBS, 100 μl of crystal violet with a concentration of 0.4% was added and incubated for 30 min at 37°C. The absorbance of the plates was then read at 570 nm with a microplate reader (Tecan, Austria) after the plates were washed again and dried.

### Biofilm formation assay

The WT strain was grown in BHI broth to an OD (600 nm) of 0.6 with chalcone or DMSO with a shaker rate of 190 rpm at 37°C (the WTΔSrtA strain was used as the positive control), and then, 10 μl of the bacterial solution was added into the 96-well flat-bottom polystyrene microtiter plates containing 290 ml BHI of broth and 3% (w/v) sucrose with or without chalcone. The mixture was incubated anaerobically under still culture conditions for 18 h at 37°C. After incubation, the liquid containing the bacteria and medium was removed, followed by the addition of 100 μl of 10% formaldehyde solution, which was then left overnight at room temperature to fix the biofilm. Subsequently, the formaldehyde was removed, and each well was stained with 100 μl of 0.1% v/v crystal violet for 30 min at room temperature. After rinsing with double distilled water and drying, 200 μl of 33% acetic acid was added to each well and all of the contents were mixed manually. The absorbance of the plates was subsequently read at 490 nm.

### Cell invasion assays

J774 cells were suspended in DMEM / HIGH GLUCOSE supplemented with 10% heat-inactivated fetal bovine serum (HI-FBS; Invitrogen), and approximately 3 × 10^5^ cells were seeded into each well of 24-well flat-bottom polystyrene microtiter plates containing 12 mm diameter coverslips overnight (37°C, 5% CO_2_). The WT strain was grown in BHI broth to an OD (600 nm) of 1.0 with chalcone or DMSO in a shaking incubator with a shaker rate of 190 rpm at 37°C; the WTΔSrtA strain was used as the positive control. The bacteria were then adjusted to an OD (600 nm) of 1.0, and 1 ml of the bacterial solution was added into each well and incubated for 60 min at 37°C. After each well was washed three times with PBS, 1 ml of DMEM / HIGH GLUCOSE supplemented with 300 μg / ml gentamicin was added and incubated for 30 min at 37°C. The J774 cells on the coverslips were then lysed in the sterile distilled water and plated onto BHI broth agar plates for CFU after washing three times with PBS. The plates were placed overnight at 37°C.

### Molecular modeling

In this work, the initial structure of SrtA was obtained from the X-ray crystallography 3D structure (PDB code: 3CI5). To obtain the starting structure of the ligand/SrtA complex for a molecular dynamics (MD) simulation, a standard docking procedure for a rigid protein and a flexible ligand was performed with AutoDock 4 (Morris et al., 2009; Hu et al., 2010). Subsequently, the molecular dynamics simulation of the complex's systems was performed, and the details of the processes of the computational biology method were described in a previous report (Dong et al., 2013; Lv et al., 2013; Niu et al., 2013).

### Binding affinity determination of ligands with proteins

In our paper, the fluorescence-quenching method was used to measure the binding constants (*K*_*A*_) of ligands with proteins. A 280-nm excitation wavelength with a 5-nm bandpass and a 345-nm emission wavelength with a 10-nm bandpass were used for the measurements. Details of the measurements were described previously (Bandyopadhyay et al., 2002; Jurasekova et al., 2009).

### Hemolysis assay

Overnight cultures of the WT strain were inoculated 1: 100 into fresh BHI broth and grown to an OD (600 nm) of 0.3 at 37°C. DMSO or different concentrations of chalcone were then added for further culture. To determine the effect of chalcone on the hemolysis activity of *S. aureus*, 1 ml of supernatant was harvested (3,000 × g, 5 min), and 100 μl of the bacterial culture supernatants were incubated with rabbit erythrocytes whose final concentration was 2.5% in PBS at 37°C for 20 min, after which the samples were centrifuged (10,000 × g, 1 min). Finally, the release of hemoglobin was measured at OD (543 nm).

To determine the effect of chalcone on hemolysis induced by the bacterial culture supernatants, the bacterial supernatants were pre-cultured with different concentrations of chalcone as described above.

### Western blotting assay

An equal volume of the bacterial culture supernatants that were harvested in the hemolysis assay was resolved using SDS-PAGE, and the proteins were transferred onto PVDF membranes. After blocking in 5% non-fat milk for 2 h, the membranes were incubated with a primary rabbit anti-Hla antibody (Sigma-Aldrich) diluted 1: 8,000 for 2 h and a horseradish peroxidase-conjugated secondary antibody (Proteintech) diluted 1: 4,000 for 2 h. The signals were visualized on a Tanon-4200 imager using Amersham ECL Western blotting detection reagents (GE Healthcare, Buckinghamshire, UK).

### Real-time RT-PCR assay

The WT strain with DMSO or various concentrations of chalcone was cultured and grown to an OD (600 nm) of 2.5 at 37°C. As described previously (Qiu et al., 2011), the total RNA from the cultured bacteria was isolated and then reverse transcribed into cDNA using the Takara RNA PCR kit (AMV), ver. 3.0 (Takara, Kyoto, Japan). According to the manufacturer's instructions, the PCR reactions were performed in 25-μl volumes using SYBR Premix Ex Taq TM (Takara). The 7000 Sequence Detection System (Applied Biosystems, Courtaboeuf, France) was used to assess the PCR amplification. All primer pairs used for this assay conformed with a previous study (Qiu et al., 2011). It is worth noting that the housekeeping gene, *gyrBRNA*, was used as an endogenous control to normalize the expressional levels between the samples.

### Live/dead and cytotoxicity assays

Hla, as a vital factor, has been shown to participate in mediating A549 cell injury and death (Bubeck and Olaf, 2008). For this reason, A549 cells were cultured and transferred into 96-well flat-bottom polystyrene microtiter plates at a density of 2 × 10^4^ cells per well in 200 μl of culture medium and incubated with the bacterial suspensions harvested above. After a 5-h incubation at 37°C, the Live/Dead (green/red) reagent (Roche) and the Cytotoxicity Detection kit (LDH, Roche) were used to assess the therapeutic effect of chalcone on A549 cells. The images of the Live/Dead cells were acquired using a confocal laser-scanning microscope, and the release of LDH was determined on a microplate reader (Tecan, Austria) at 490 nm.

### Animal experiments

The mice (6- to 8-week-old-female C57BL/6J) used for the animal experiments were obtained from the Experimental Animal Center of Jilin University. The animal experiments were approved by and conducted in accordance with the guidelines of the Animal Care and Use Committee of Jilin University. Overnight cultures of *S. aureus* were inoculated 1: 100 into fresh BHI broth and grown to an OD (600 nm) of 0.6 at 37°C. After centrifugal collection (3,000 × g for 5 min) and washing three times with PBS, the bacteria were resuspended in PBS to the required concentration for survival studies. Bacteria (4 × 10^8^ CFUs) were dropped into the left nare of the mice. To investigate the therapeutic effect of chalcone, the mice were treated with a subcutaneous injection of chalcone of 150 mg/kg after infection with *S. aureus* and then at 12-h intervals thereafter. The control mice were treated with equal volumes of DMSO simultaneously. The mortality analysis was monitored after 96 h, with ten mice contained in each experimental group.

### Statistical analysis

The statistical significance of the treated and control group were assessed using the log-rank tests for the survival curves, and for other assays, a Student's *t*-test was used. The differences were analyzed using SPSS 13.0 (SPSS Inc., Chicago, IL, USA) statistical software, and the differences were considered statistically significant when *P* < 0.05. The data are presented as the mean ± SD.

## Results

### Chalcone inhibits the activity of SrtA and the expression of Hla

Chalcone (Figure 1A), one of the effective components of herbal plants, has been identified to be a remarkable inhibitor against bacteria virulence factors (Wallockrichards et al., 2015; Li et al., 2016). In this study, chalcone was shown to be a potential inhibitor against *S. aureus* SrtA and Hla. A FRET assay was used to determine the inhibitory activity of chalcone against SrtA, and the result indicated that the inhibition of SrtA activity was significant in the presence of various concentrations of chalcone (Figure 1A). We determined that the half maximal inhibitory concentration (IC_50_) of chalcone was 53.15 μM. It is worth noting that the minimum inhibitory concentration (MIC) of chalcone against the tested *S. aureus* strain was >4,864 μM. Meanwhile, the growth of *S. aureus* USA 300 was not visibly affected by chalcone at concentrations sufficient to inhibit SrtA (Figure 1B).

**Figure 2:**
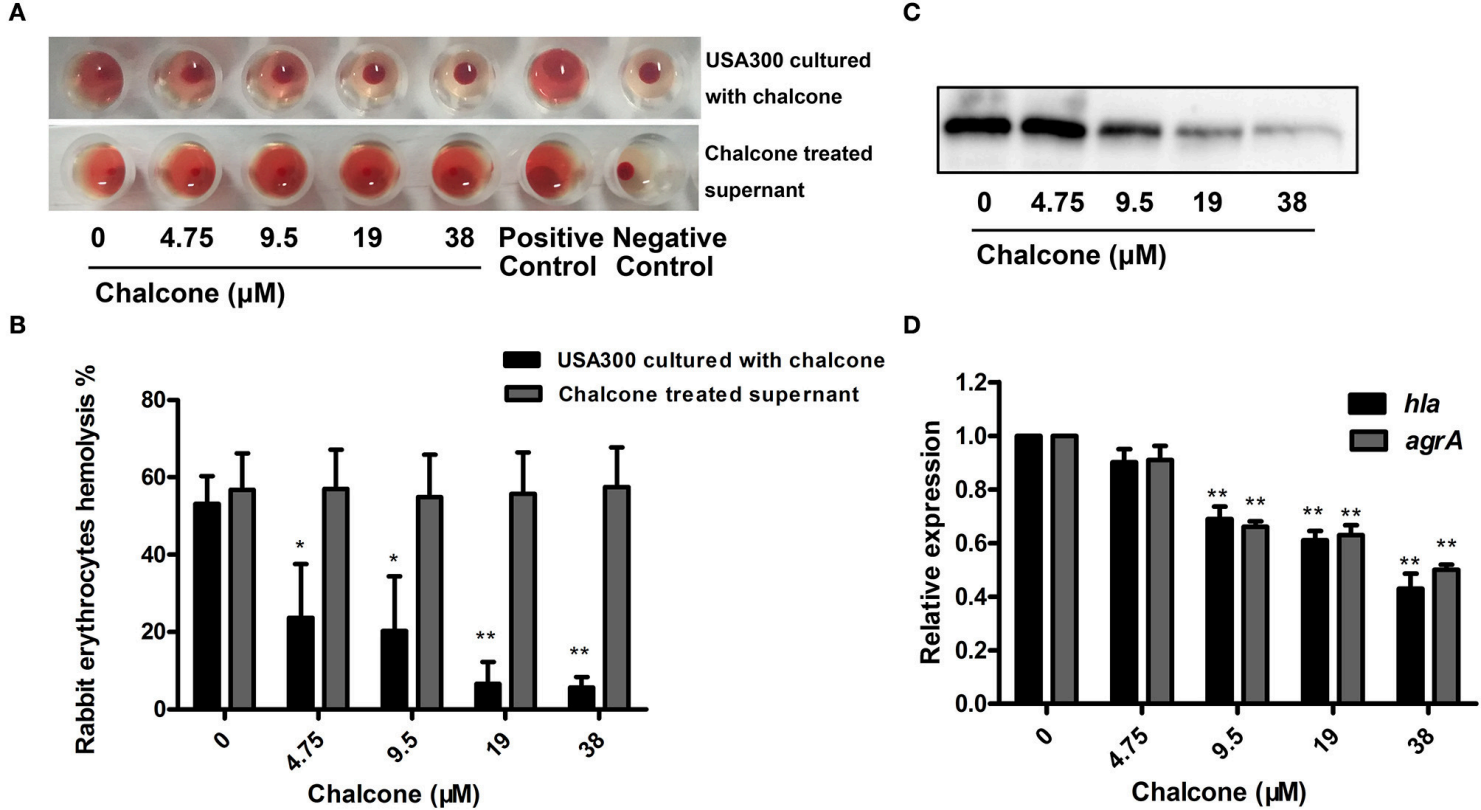
Chalcone inhibits *S. aureus* Hla expression. **(A)** The hemolysis activity of culture supernatants by *S. aureus* co-cultured with chalcone. **(B)** Bacterial supernatants pre-incubated with chalcone and then the hemolysis activity was determined. **(C)** Western blotting assay to detect the Hla expression in the bacterial culture supernatants by *S. aureus* co-cultured with or without chalcone. **(D)** The relative gene expression of the *hla* gene and the *agrA* gene in *S. aureus* exposed to chalcone at different concentrations. Three independent experiments were performed to obtain stable results. *P < 0.05 vs. the WT group and **P < 0.01 vs. the WT group.

Furthermore, a hemolysis assay was used to determine the effect of chalcone on the hemolysis activity of the bacterial cultural supernatants. The results showed that chalcone at the concentrations tested in this study could significantly decrease the hemolysis activity of *S. aureus* in a dose-dependent manner when co-cultured with USA 300 strain (Figures 2A,B). Notably, minimal hemolysis was detected when the strain was cultured with 38 μM chalcone, and the IC_50_ value for chalcone-induced inhibition of Hla was 17.63 μM. In addition, pre-incubation with chalcone had almost no effect on the hemolytic activities of the bacterial culture supernatants (Figures 2A,B), implying that chalcone-induced inhibition of Hla might act by means of blocking its expression. To verify this, a Western blotting assay with the bacterial culture supernatants harvested above and a real-time RT-PCR assay were performed. In line with our conjecture, the Hla levels in the supernatants were reduced in a dose-independent manner (Figure 2C), and under our experimental conditions, the transcription levels of the *hla* gene and the *agrA* gene in USA 300 were both down-regulated by chalcone in a dose-independent manner (Figure 2D). Taken together, our results established that chalcone was able to effectively inhibit SrtA activity and the expression of Hla while exerting little antimicrobial activity, implying that chalcone could represent an effective anti-infective agent for *S. aureus* infection.

### Chalcone influences the SpA display in intact cells

**Figure 3:**
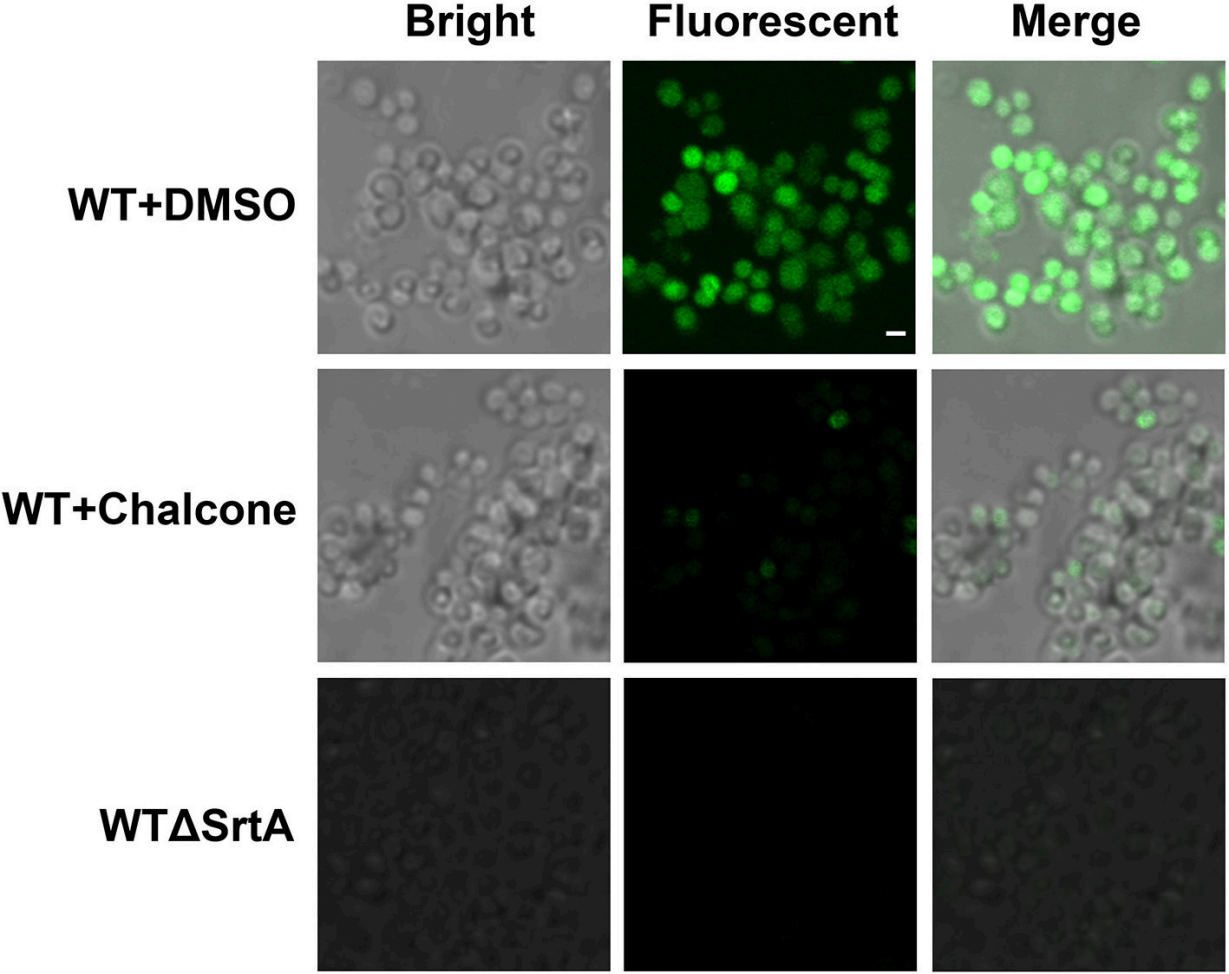
Effects of chalcone on SpA display in *S. aureus*. A confocal laser-scanning microscope was used to view the binding of FITC-labeled Ig to SpA. The strength of the green color represents the amount of the SpA anchored to the surface of bacteria. Scale bar, 1 μm.

*Staphylococcus aureus* SrtA anchors many different surface proteins in the cell wall, including SpA, a multifunctional molecule responsible for binding the Fcγ portion of host immunoglobulins (Falugi et al., 2013). Therefore, SpA is vital for immune evasion, and because of its function, a change in the amount of SpA in the cell wall after the addition of chalcone requires investigation. In this assay, *S. aureus* cultured with or without chalcone was stained with FITC-labeled goat anti-rabbit IgG, which made the cells display a green color whose strength was determined by the amount of SpA in the cell wall. With the aid of confocal laser-scanning microscope, the content variation of SpA in the cell wall was investigated. The result showed that the WTΔSrtA strain, used as a control, displayed much less SpA on its surface compared with the WT strain, and as expected, 76 μM chalcone present in the cell culture caused significant reductions in the SpA display (Figure 3). These consequences indicated that chalcone was capable of disturbing the assembly of sortase-mediated SpA in the cell wall.

### Chalcone reduces the adherence of *S. aureus* to fibronectin

**Figure 4:**
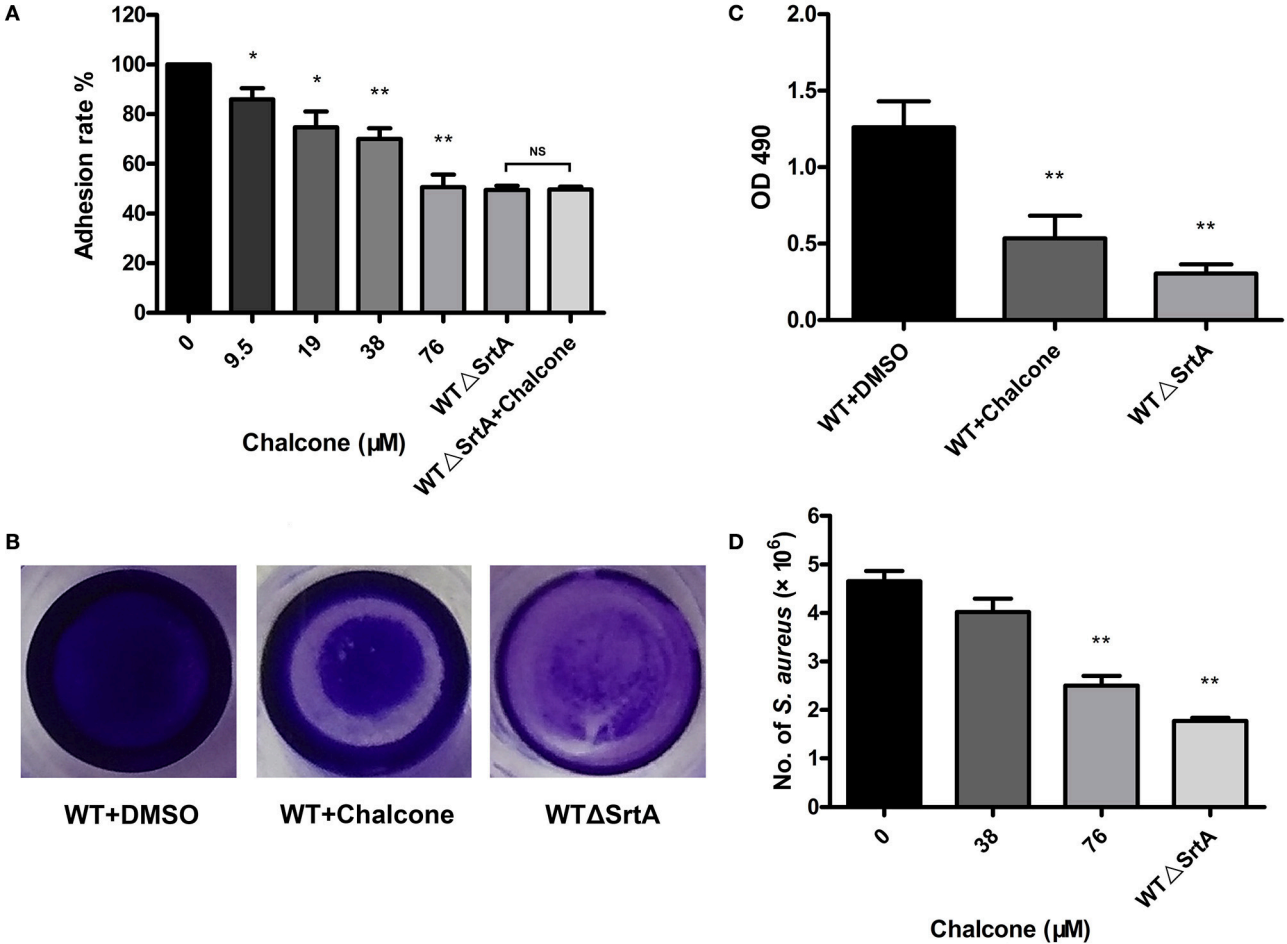
Chalcone reduces the adhesion of *S. aureus* to Fn, biofilm formation and *S. aureus* invasion. **(A)** Adhesion rate of *S. aureus* to Fn in the presence of different concentrations of chalcone. The WTΔSrtA strain was incubated with 76 μM chalcone. **(B)** Photographs of biofilms grown in 96-well flat-bottom polystyrene microtiter plates. **(C)** Quantification of the biofilm mass. **(D)** Chalcone weakened *S. aureus* invasion. Bacteria and J774 cells were cultured with chalcone at different concentrations at 37°C for 1 h, followed by gentamicin addition and incubation at 37°C for 30 min, after which number of intracellular bacteria was determined. Three independent experiments were performed to obtain stable results. *P < 0.05 vs. the WT group, **P < 0.01 vs. the WT group and NS represents no significance.

In the previous study, we have drawn the conclusion that chalcone can visibly inhibit the SrtA-catalyzed transpeptidation at relatively low concentrations. The fibronectin-binding proteins FnBPA and FnBPB are LPXTG proteins expressed by *S. aureus*. Fibronectin (Fn) is regarded as a bridge between the bacterial adhesion FnBP and the mammalian cell integrin, accelerating the process of phagocytosis, thereby stimulating the internalization of bacteria; consequently, Fn and FnBPs play a vital role in the process of invading host cells (Foster and Höök, 1998; Roche et al., 2003). Because FnBPs anchored by *S. aureus* SrtA can make bacteria invasive, if their display in the cell wall is blocked by weakening SrtA activity, then bacterial invasion would be decreased. For this reason, we employed Fn-binding assays to determine if chalcone could reduce the adherence of *S. aureus* to Fn. As expected, the WTΔSrtA strain had a minimum adhesion rate to Fn owing to the damage to its Fn-binding function and no inhibition was observed in the samples treated with chalcone (Figure 4A). However, when the WT strain was treated with chalcone at different concentrations, the adhesion rates decreased in turn, respectively, indicating that the effect occurred in a dose-dependent manner. The variance between the 38 μM chalcone group and the WT group was a very significant difference, as was the 76 μM chalcone group (Figure 4A).

### Chalcone reduced biofilm formation

Bacteria enhance their ability to resist antibiotics and other antimicrobial agents by embedding in biofilms (Pugliese and Favero, 2002; Cerca et al., 2005; Høiby et al., 2010). Biofilm phenotypes are promoted by the FnBPs, the major cell wall autolysin or the *icaADBC*-encoded polysaccharide intercellular adhesin/poly-N-acetylglucosamine (PIA/PNAG) (O'Neill et al., 2007, 2008; Clarissa Pozzi et al., 2012). To investigate whether chalcone hindered biofilm formation, *S. aureus* biofilm formation was determined with or without chalcone. After dye treatment, the WTΔSrtA group was much more lightly stained than the WT group, and the visible difference between the biofilms formed under the conditions of untreated and treated with 76 μM chalcone was quite significant (Figure 4B). These results were in accordance with those of the quantitative analysis (Figure 4C). In summary, chalcone reduced biofilm formation, suggesting that the inhibitory effect of chalcone on *S. aureus* SrtA had occurred.

### Chalcone weakened the *S. aureus* invasion

SrtA function is normally important for the display of sortase-mediated surface proteins, thus enabling bacteria to invade the host cells. To examine how chalcone affects *S. aureus* invasion, J774 cells were cultured with bacteria and chalcone at different concentrations. Not surprisingly, the numbers of bacteria entering the cells in the WTΔSrtA group showed a significant decrease compared with the WT group (Figure 4D). Consistent with the hypothesis, the addition of a dose of chalcone to the experimental system significantly decreased the quantity of bacteria, indicating that chalcone weakened the *S. aureus* invasion by inhibiting SrtA activity (Figure 4D).

### Molecular dynamics simulation for SrtA-chalcone

**Figure 5:**
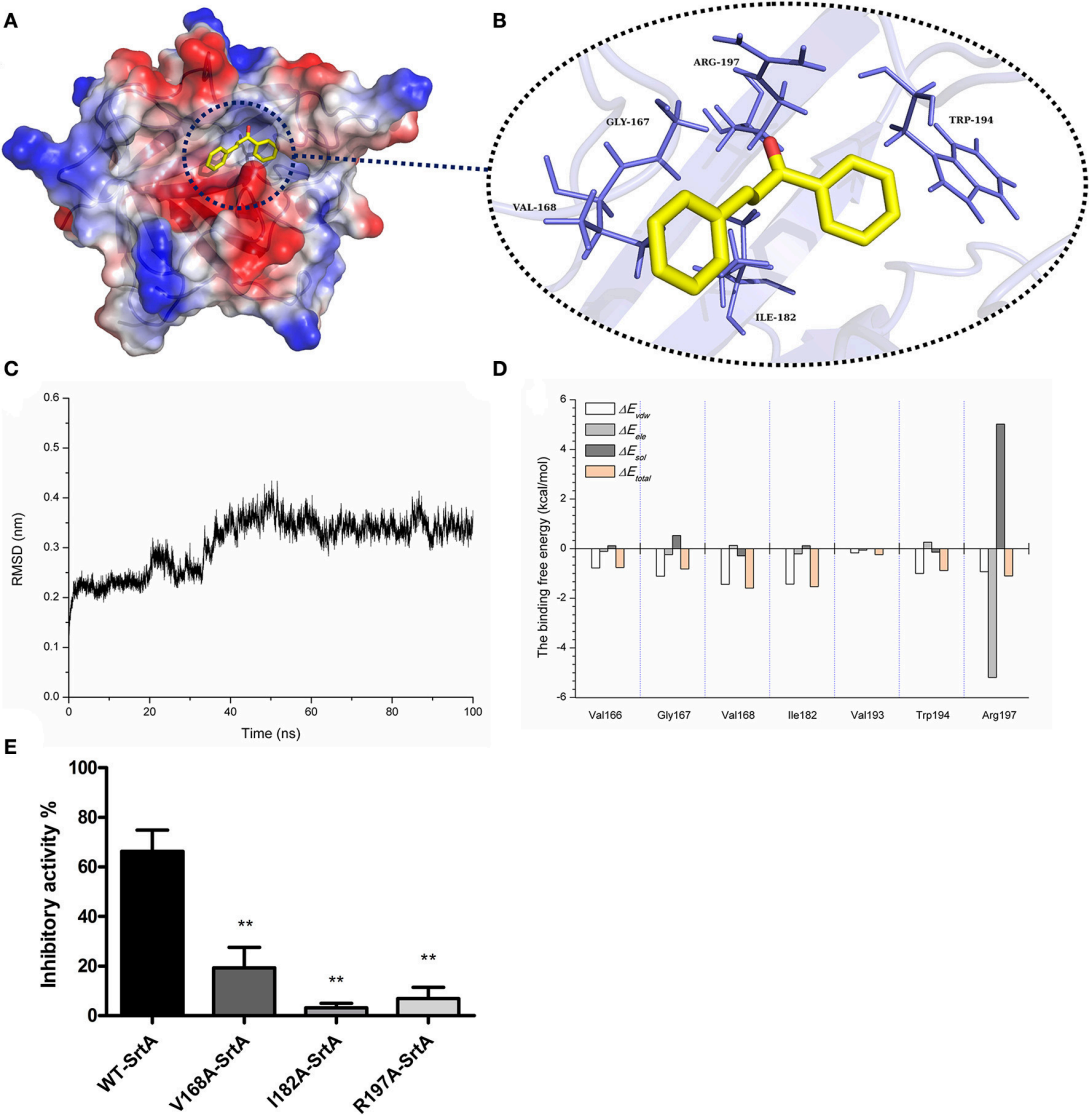
The 3D structural determination of a SrtA with chalcone complex by a molecular modeling method and the inhibitory effects of chalcone against WT-SrtA and SrtA mutants. **(A)** The structure of SrtA-chalcone. **(B)** The interaction of chalcone with the key residues of SrtA. **(C)** The RMSD displayed by the backbone atoms of the protein during MD simulations of SrtA-Chalcone is presented. **(D)** Decomposition of the binding energy on a per-residue basis at the binding sites of the SrtA-chalcone complex. **(E)** WT-SrtA and SrtA mutants (V168A-SrtA, I182A-SrtA and R197A-SrtA) were incubated with 76 μM chalcone, and the catalytic activity of the recombinant SrtA was determined as described in Figure 1A. **P < 0.01 vs. the WT group.

Through the computational biology method, the potential binding mode of chalcone with SrtA in the active site was explored in this study. The chalcone was bound to SrtA, and according to the binding mode given (Figures 5A,B), it was clear that chalcone could bind to SrtA via Van der Waals and electrostatic interactions. During the time course of the simulation, chalcone could localize to the catalytic pocket of SrtA (residue 160–200). In detail, the binding model of chalcone with SrtA revealed that chalcone could form strong interactions with Val166, Gly167, Val168, Ile182, Val193, and Arg197, respectively. The complex was found to reach equilibrium at 100 ns based on the analysis of the root-mean-square deviations (RMSD) of the backbone C_α_ atoms (Figure 5C), which indicated that the complex system has reached equilibrium.

To explore the energy contributions from the residues of the binding sites in the SrtA- chalcone complex, the energy decomposition was calculated for the SrtA-chalcone complex system. Val168, Ile182 and Arg197 had a strong total binding energy contribution, with a Δ*E*_*total*_ of < -1.0 kcal/mol (Figure 5D). In addition, residues Gly167 and Trp194 also had the appreciable total binding energy contribution, with a Δ*E*_*total*_ of < -0.8 kcal/mol. These results suggested that these five residues were key residues for chalcone.

To confirm these theoretical results, the total binding free energy for the SrtA- chalcone complex and their detailed energy contributions calculated according to the MM-PBSA approach are summarized in Table 2. According to the calculation results, the binding free energy, Δ*G*_*bind*_, of the interaction between chalcone and the protein decreased in the following order: WT-SrtA > V168A-SrtA > R197A-SrtA > I182A-SrtA, which means that WT-SrtA had the strongest ability to bind to chalcone. Using fluorescence spectroscopy quenching, we measured the Δ*G*_*bind*_ and the number of binding sites between chalcone and the three mutants, and these results were highly consistent with those obtained by computational methods (Table 2). To further validate the simulation results, three mutants, V168A-SrtA, I182A-SrtA, and R197A-SrtA, were constructed for the FRET assays. As expected, when compared with WT-SrtA, chalcone was significantly less sensitive for these three mutants (Figure 5E). These results indicated that the information generated by the MD simulation on the SrtA-Chalcone complex was reliable. Due to the binding of inhibitor, chalcone, with the activity region (residues of Gly167, Val168, Ile182, Cys184, Trp194, and Arg197), the biology activity of SrtA was inhibited.

**Table 2:** The binding free energy (kcal/mol) of WT-Chalcone, V168A-Chalcone, I182A-Chalcone, and R197A-Chalcone systems based on computational method and the values of the binding constants (*K*_*A*_) based on the fluorescence spectroscopy quenching.

**Proteins**	**WT-SrtA**	**V168A**	**I182A**	**R197A**
The binding energy	−7.82 ± 0.9	−5.5 ± 0.4	−4.7 ± 0.5	−5.1 ± 0.6
*K*_A_ (1 × 10^4^) L·mol^−1^	6.1 ± 0.5	3.8 ± 0.4	4.1 ± 0.8	4.0 ± 0.7

### Chalcone protects A549 cells from Hla-mediated injury

**Figure 6:**
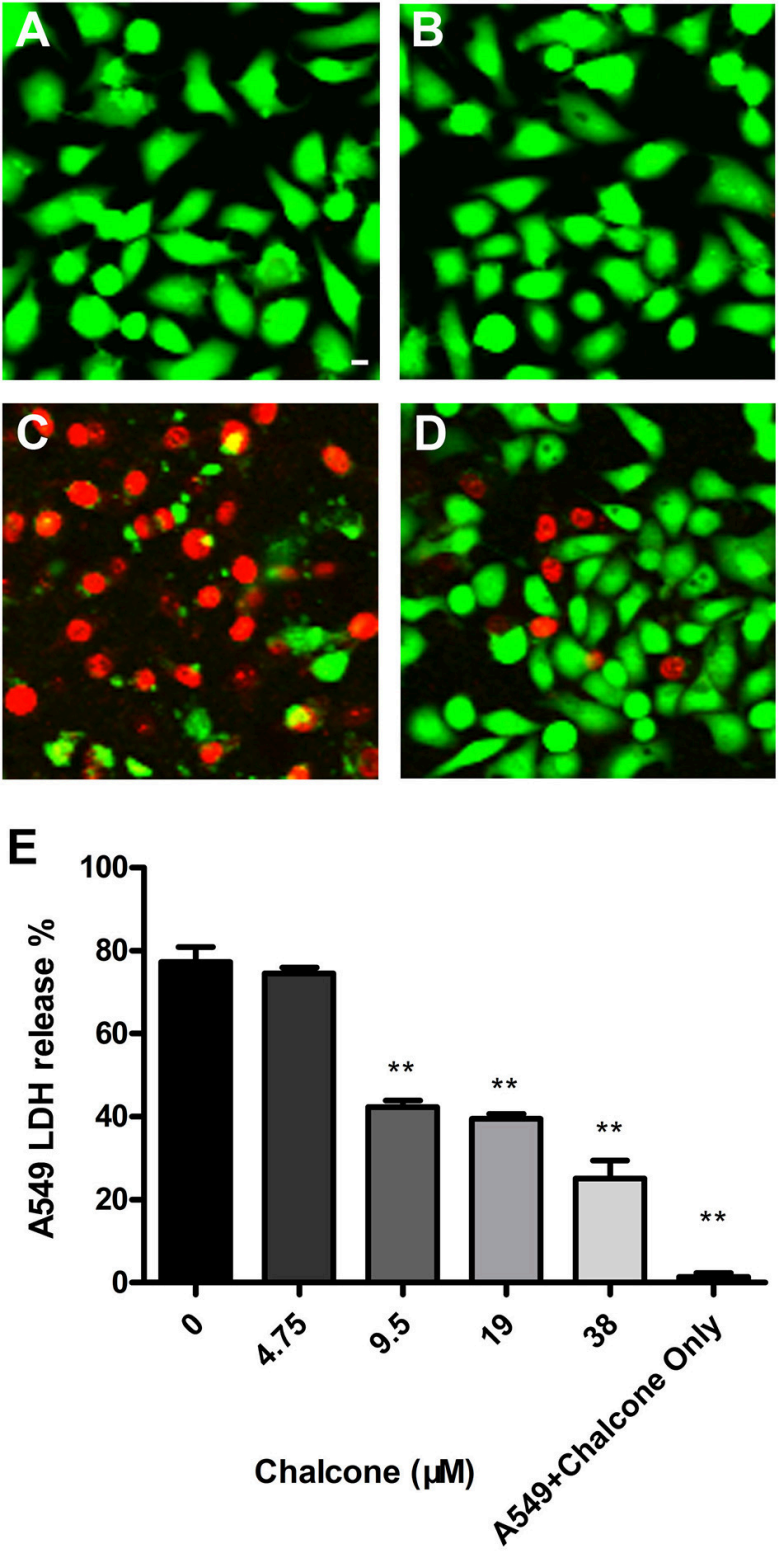
Chalcone protects A549 cells from injury mediated by *S. aureus* Hla. Live/Dead reagent-A549 cells were observed with fluorescent imaging. **(A)** The untreated A549 cells. **(B)** A549 cells treated with 38 μM chalcone only. **(C)** A549 cells infected with *S. aureus* culture supernatants harvested in the absence of chalcone. **(D)** A549 cells treated with *S. aureus* culture supernatants harvested in the presence of 38 μM chalcone. **(E)** The LDH release by A549 cells treated with *S. aureus* culture supernatants harvested previously and 38 μM chalcone only, respectively. Scale bar, 10 μm. **P < 0.01 vs. the WT group.

In our co-culture system, the potential protective effect of chalcone on the Hla-induced injury of A549 cells was determined. In the Live/Dead assay, uninfected cells retained green fluorescence (Figure 6A), and inversely, injured cells retained red fluorescence. As expected, 38 μM chalcone showed an obvious reduction in cell injury, with almost no cytotoxic effect observed in A549 cells (Figures 6B–E). Simultaneously, the LDH assay showed that treatment with chalcone could significantly decrease the release of LDH into the supernatants in a dose-independent manner compared with the control group, indicating less cell death (Figure 6E). Above all, the results showed that chalcone had a protective effect on Hla–mediated A549 cell injury.

### Chalcone protected mice from fatal *S. aureus* infection

**Figure 7:**
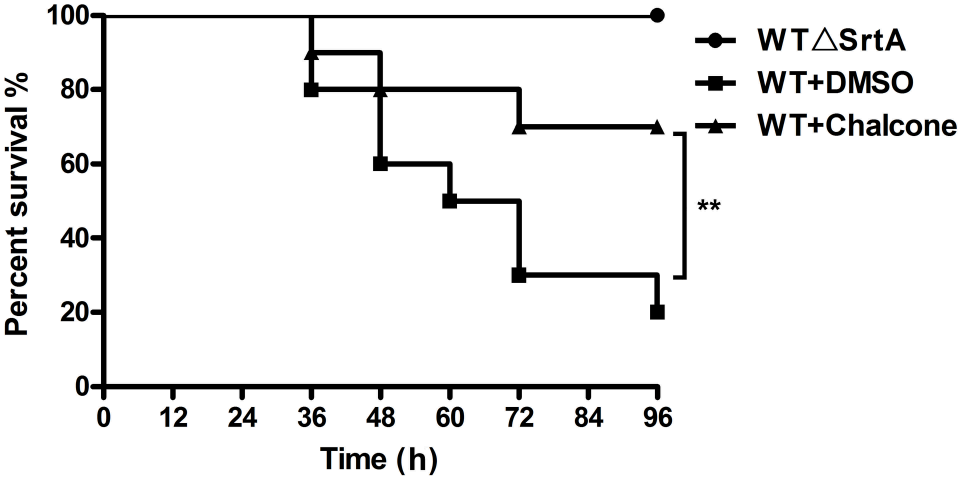
Effects of chalcone on survival rates after 96 hours in C57BL/6J mice. Mice were infected with 4 × 10^8^ CFUs of *S. aureus* and the WTΔSrtA strain. Treatment with chalcone (150 mg/kg, twice a day) was initiated after infection. **P < 0.01 vs. the WT group.

To investigate the effect of chalcone on the survival rate of mice inoculated with *S. aureus*, we performed survival experiments. Ninety-six hours after infection with 4 × 10^8^ CFUs of bacteria, only 20% of the WT-infected mice survived, in contrast to the WTΔSrtA group in which the survival rate was 100% (Figure 7). In the chalcone-treated group, the survival rate was significantly higher than in the group without treatment (Figure 7). The death of mice in the WT+chalcone group occurred only at 36, 48, and 72 h in the whole process of infection (Figure 7). These findings suggested that chalcone could prolong survival of the mice in *S. aureus*–induced infection.

## Discussion

*Staphylococcus aureus* is a major human pathogen and is also a leading cause of sepsis and infective endocarditis. The continuous spread of antibiotic-resistant strains, such as MRSA and VRSA makes treatment very difficult (Lowy, 1998; Petti and Fowler, 2003; Menichetti, 2005), and new valid strategies are greatly needed to address this severe situation. The virulence factors of bacteria, such as adhesins, invasins and toxins, among others, facilitate infection by evading the immune response and leading to colonization, spread and tissue damage (Lowy, 1998; Foster, 2005; Rooijakkers et al., 2005). In addition, previous studies have demonstrated that the vast majority of these various virulence factors are nonessential for bacterial survival and are crucial in the process of targeting host cells and causing disease (Lowy, 1998; Berube and Bubeck, 2013). Some previous studies have also demonstrated that *S. aureus* lacking the genes encoding SrtA or Hla will show a weakened bacterial virulence (Albus et al., 1991; Mcelroy et al., 1999; Mazmanian et al., 2000; Wardenburg et al., 2007). For this reason, therapeutic agents targeting virulence factors, such as SrtA and Hla, which do not threaten survival, may not lead to the development of resistance as quickly as conventional antibiotics typically do, and this will have important implications for *S. aureus* infection. Some agents targeting *S. aureus* SrtA or Hla have been found previously (Liu et al., 2015; Zhou et al., 2015; Zhang et al., 2016). However, as these compounds are targeted only at one kind of virulence factor, the limitations for clinical treatment will certainly exist. A hypothesis was then proposed as to whether we could find an agent targeting SrtA and Hla simultaneously to produce a better therapeutic effect.

The scope of screening for SrtA and Hla inhibitors was wide, involving natural, synthetic and high-throughput screening methodologies. We sought to find inhibitors from natural small molecule compounds, from which chalcone, of the flavonoid class of natural products (Figure 1A), with relatively simple structures and various functions (Sahu et al., 2012), was discovered. First, a FRET assay and a hemolysis assay were used to identify the inhibition of chalcone on SrtA and Hla, respectively. After incubating *S. aureus* with chalcone at a certain concentration, the SrtA catalytic activity, hemolysis ratio and Hla expression in the bacterial culture supernatant were all decreased (Figures 1A, 2A–C). Additionally, chalcone indeed affected the transcriptional levels of the *hly* gene and the *agrA* gene (Figure 2D), indicating that the decreased expression of Hla might be caused by the decreased expression of the genes. In addition, the decreased expression of Hla conferred significant protection against Hla-mediated A549 cell injury (Figure 6). We also found that the inhibitory effect of chalcone against SrtA reduced the SpA display in the cell wall (Figure 3), decreased the adherence of *S. aureus* to fibronectin (Figure 4A) and resulted in the lower biofilm formation (Figures 4B,C). In addition, as far as we know, our study is the first to use a cell invasion assay to evaluate the inhibitory activity of a natural compound on SrtA, which showed that the quantity of bacterial entry into cells was significantly decreased by chalcone (Figure 4D). To explore the interaction mechanism between chalcone and SrtA, a molecular dynamics simulation for a SrtA-chalcone complex system was carried out. By means of molecular dynamics simulation, it was found that chalcone could localize to the catalytic pocket of SrtA (residues 160–200), which is very close to the binding site of substrate. Due to the binding of chalcone to SrtA, the binding of substrate to SrtA was blocked, leading to the loss of biological activity of SrtA (Figure 5). It is worth mentioning that just at a much lower concentration than the MIC, chalcone could significantly stop *S. aureus* SrtA-mediated transpeptidation and the hemolysis activity of Hla *in vitro* by inhibiting SrtA activity and the expression of Hla, respectively, instead of killing bacteria directly (Figure 1B), which means a less selective pressure for bacteria and a lower risk of drug resistance. More importantly, a C57BL/6J mouse model was established to determine the therapeutic effect of chalcone *in vivo*, and treatment with chalcone significantly attenuated the virulence of *S. aureus* and protected mice from infection caused by the bacteria (Figure 7). To the best of our knowledge, this is the first report of the discovery and proof of an inhibitor targeting *S. aureus* SrtA and Hla simultaneously, and thus, this kind of inhibitor can be considered as a novel promising candidate against *S. aureus* infection.

In conclusion, our study discovered that the natural small compound chalcone could effectively inhibit *S. aureus* SrtA activity and the expression of Hla by occupying the sites of the enzyme to prevent anchoring of surface proteins and decreasing transcription levels of the *hla* gene and the *agrA* gene, thereby attenuating *S. aureus* virulence both *in vitro* and *in vivo*. These findings could be the foundation for further design of novel anti-infection agents, and a SrtA-Hla-centered strategy has emerged accordingly, thus making a contribution for opening a new horizon for the treatment of *S. aureus* infection.

## Author contributions

JW and XN conceived and designed the experiments. BZ, ZT, XL, and GL performed the experiments. XD contributed reagents/materials/analysis tools. JW, BZ, and XD wrote the paper.

### Conflict of interest statement

The authors declare that the research was conducted in the absence of any commercial or financial relationships that could be construed as a potential conflict of interest.

## References

[B1] Albus A. Arbeit R. D. Lee J. C. (1991). Virulence of *Staphylococcus aureus* mutants altered in type 5 capsule production. Infect. Immun. 59, 1008–1014. 1847696 10.1128/iai.59.3.1008-1014.1991 PMC258360

[B2] Bandyopadhyay S. Valder C. R. Huynh H. G. Ren H. Allison W. S. (2002). The beta G156C substitution in the F1-ATPase from the thermophilic Bacillus PS3 affects catalytic site cooperativity by destabilizing the closed conformation of the catalytic site. Biochemistry 41, 14421–14429. 10.1021/bi026243g 12450409

[B3] Berube B. J. Bubeck W. J. (2013). *Staphylococcus aureus* α-Toxin: nearly a century of intrigue. Toxins (Basel) 5, 1140–1166. 10.3390/toxins5061140 23888516 PMC3717774

[B4] Bubeck W. J. Olaf S. (2008). Vaccine protection against *Staphylococcus aureus* pneumonia. J. Exp. Med. 205, 287–294. 10.1084/jem.20072208 18268041 PMC2271014

[B5] Cerca N. Martins S. Cerca F. Jefferson K. K. Pier G. B. Oliveira R. (2005). Cerca, N. Comparative assessment of antibiotic susceptibility of coagulase negative staphylococci in biofilm versus planktonic culture as assessed by bacterial enumeration or rapid XTT colorimetry. J. Antimicrob. Chemother. 56, 331–336. 10.1093/jac/dki217 15980094 PMC1317301

[B6] Clancy K. W. Melvin J. A. Mccafferty D. G. (2010). Sortase transpeptidases: insights into mechanism, substrate specificity, and inhibition. Biopolymers 94, 385–396. 10.1002/bip.21472 20593474 PMC4648256

[B7] Clarissa Pozzi E. M. W. Justine Rudkin K. Carolyn Schaeffer R. Amanda Lohan J. Pin T. Brendan Loftus J.. (2012). Methicillin resistance alters the biofilm phenotype and attenuates virulence in *Staphylococcus aureus* device-associated infections. PLoS Pathog. 8:e1002626. 10.1371/journal.ppat.1002626 22496652 PMC3320603

[B8] Coates R. Moran J. Horsburgh M. J. (2014). Staphylococci: colonizers and pathogens of human skin. Future Microbiol. 9, 75–91. 10.2217/fmb.13.145 24328382

[B9] Dinges M. M. Orwin P. M. Schlievert P. M. (2000). Exotoxins of *Staphylococcus aureus*. Clin. Microbiol. Rev. 13, 16–34, table of contents. 10.1128/CMR.13.1.16-34.2000 10627489 PMC88931

[B10] Dong J. Qiu J. Zhang Y. Lu C. Dai X. Wang J.. (2013). Oroxylin A inhibits hemolysis via hindering the self-assembly of α-hemolysin heptameric transmembrane pore. PLoS Comput. Biol. 9:e1002869. 10.1371/journal.pcbi.1002869 23349625 PMC3547825

[B11] Falugi F. Kim H. K. Missiakas D. M. Schneewind O. (2013). Role of protein a in the evasion of host adaptive immune responses by *Staphylococcus aureus*. MBio 4:e00575. 10.1128/mBio.00575-13 23982075 PMC3760252

[B12] Fischetti V. A. Pancholi V. Schneewind O. (1990). Conservation of a hexapeptide sequence in the anchor region of surface proteins from Gram-positive cocci. Mol. Microbiol. 4, 1603–1605. 10.1111/j.1365-2958.1990.tb02072.x 2287281

[B13] Foster T. J. (2005). Immune evasion by staphylococci. Nat. Rev. Microbiol. 3, 948–958. 10.1038/nrmicro1289 16322743

[B14] Foster T. J. Höök M. (1998). Surface protein adhesins of *Staphylococcus aureus*. Trends Microbiol. 6:484. 10.1016/S0966-842X(98)01400-0 10036727

[B15] Gould I. M. (2013). Treatment of bacteraemia: meticillin-resistant *Staphylococcus aureus* (MRSA) to vancomycin-resistant *S. aureus* (VRSA). Int. J. Antimicrob. Agents 42, 23–30. 10.1016/j.ijantimicag.2013.04.006 23664580

[B16] Høiby N. Bjarnsholt T. Givskov M. Molin S. Ciofu O. (2010). Antibiotic resistance of bacterial biofilms. Int. J. Antimicrob. Agents 35, 322–332. 10.1016/j.ijantimicag.2009.12.011 20149602

[B17] Hu R. Barbault F. Maurel F. Delamar M. Zhang R. (2010). Molecular dynamics simulations of 2-Amino-6-arylsulphonylbenzonitriles analogues as HIV inhibitors: interaction modes and binding free energies. Chem. Biol. Drug Des. 76, 518–526. 10.1111/j.1747-0285.2010.01028.x 20942836

[B18] Ippolito G. Leone S. Lauria F. N. Nicastri E. Wenzel R. P. Ippolito G. (2010). Methicillin-resistant *Staphylococcus aureus*: the superbug. Int. J. Infect. Dis. 14(Suppl. 4), S7–S11. 10.1016/j.ijid.2010.05.003 20851011

[B19] Jurasekova Z. Marconi G. Sanchez-Cortes S. Torreggiani A. (2009). Spectroscopic and molecular modeling studies on the binding of the flavonoid luteolin and human serum albumin. Biopolymers 91, 917–927. 10.1002/bip.21278 19603495

[B20] Levy S. B. Fitzgerald G. B. Macone A. B. (1976). Spread of antibiotic-resistant plasmids from chicken to chicken and from chicken to man. Nature 260, 40–42. 10.1038/260040a0 772441

[B21] Li H. Chen Y. Zhang B. Niu X. Song M. Luo Z.. (2016). Inhibition of sortase A by chalcone prevents *Listeria monocytogenes* infection. Biochem. Pharmacol. 106, 19–29. 10.1016/j.bcp.2016.01.018 26826492

[B22] Lin W. Bi C. Cai H. Liu B. Zhong X. Deng X. (2015). The therapeutic effect of chlorogenic acid against *Staphylococcus aureus* infection through sortase A inhibition. Front. Microbiol. 6:1031 10.3389/fmicb.2015.01031 26528244 PMC4608362

[B23] Liu S. Zhou X. Li W. Zhang H. Zhang B. Li G. (2015). Diosmetin inhibits the expression of alpha-hemolysin in *Staphylococcus aureus*. Antonie Van Leeuwenhoek 108:383 10.1007/s10482-015-0491-6 26021482

[B24] Lowy F. D. (1998). *Staphylococcus aureus* infections. N. Eng. J. Med. 339, 2026–2027. 9882209

[B25] Lv Z. Wang H. S. Niu X. D. (2013). Molecular dynamics simulations reveal insight into key structural elements of aaptamines as sortase inhibitors with free energy calculations. Chem. Phys. Lett. 585, 171–177. 10.1016/j.cplett.2013.08.097

[B26] Mazmanian S. K. Liu G. Jensen E. R. Lenoy E. Schneewind O. (2000). *Staphylococcus aureus* sortase mutants defective in the display of surface proteins and in the pathogenesis of animal infections. Proc. Natl. Acad. Sci. U.S.A. 97, 5510–5515. 10.1073/pnas.080520697 10805806 PMC25859

[B27] Mcelroy M. C. Harty H. R. Hosford G. E. Boylan G. M. Pittet J. F. Foster T. J. (1999). Alpha-toxin damages the air-blood barrier of the lung in a rat model of *Staphylococcus aureus*-induced pneumonia. Infect. Immun. 67:5541. 10496947 10.1128/iai.67.10.5541-5544.1999 PMC96922

[B28] Menichetti F. (2005). Current and emerging serious Gram-positive infections. Clin. Microbiol. Infect. 11(Suppl. S3), 22–28. 10.1111/j.1469-0691.2005.01138.x 15811021

[B29] Morris G. M. Huey R. Lindstrom W. Sanner M. F. Belew R. K. Goodsell D. S.. (2009). AutoDock4 and AutoDockTools4: automated docking with selective receptor flexibility. J. Comput. Chem. 30, 2785–2791. 10.1002/jcc.21256 19399780 PMC2760638

[B30] Niu X. Qiu J. Wang X. Gao X. Dong J. Wang J.. (2013). Molecular insight into the inhibition mechanism of cyrtominetin to α-hemolysin by molecular dynamics simulation. Eur. J. Med. Chem. 62, 320–328. 10.1016/j.ejmech.2013.01.008 23376250

[B31] O'Neill E. Pozzi C. Houston P. Humphreys H. Robinson D. A. Loughman A.. (2008). A novel *Staphylococcus aureus* biofilm phenotype mediated by the fibronectin-binding proteins, FnBPA and FnBPB. J. Bacteriol. 190, 3835–3850. 10.1128/JB.00167-08 18375547 PMC2395027

[B32] O'Neill E. Pozzi C. Houston P. Smyth D. Humphreys H. Robinson D. A.. (2007). Association between methicillin susceptibility and biofilm regulation in *Staphylococcus aureus* isolates from device-related infections. J. Clin. Microbiol. 45:1379. 10.1128/JCM.02280-06 17329452 PMC1865887

[B33] Petti C. A. Fowler V. G. Jr. (2003). *Staphylococcus aureus* bacteremia and endocarditis. Cardiol. Clin. 21:219. 10.1016/S0733-8651(03)00030-4 12874895

[B34] Pugliese G. Favero M. S. (2002). biofilms: survival mechanisms of clinically relevant microorganisms. Clin. Microbiol. Rev. 15:167 10.1128/CMR.15.2.167-193.2002 11932229 PMC118068

[B35] Qiu J. Li H. Meng H. Hu C. Li J. Luo M.. (2011). Impact of luteolin on the production of alpha-toxin by *Staphylococcus aureus*. Lett. Appl. Microbiol. 53:238. 10.1111/j.1472-765X.2011.03098.x 21671964

[B36] Ragle B. E. Karginov V. A. Bubeck W. J. (2010). Prevention and treatment of *Staphylococcus aureus* pneumonia with a -cyclodextrin derivative. Antimicrob. Agents Chemother. 54, 298–304. 10.1128/AAC.00973-09 19805564 PMC2798498

[B37] Roche F. M. Massey R. Peacock S. J. Day N. P. Visai L. Speziale P.. (2003). Characterization of novel LPXTG-containing proteins of *Staphylococcus aureus* identified from genome sequences. Microbiology 149(Pt 3), 643. 10.1099/mic.0.25996-0 12634333

[B38] Rooijakkers S. H. M. Kessel K. P. M. V. Strijp J. A. G. V. (2005). Staphylococcal innate immune evasion. Trends Microbiol. 13:596. 10.1016/j.tim.2005.10.002 16242332

[B39] Sahu N. K. Balbhadra S. S. Choudhary J. Kohli D. V. (2012). Exploring pharmacological significance of chalcone scaffold: a review. Curr. Med. Chem. 19, 209–225. 10.2174/092986712803414132 22320299

[B40] Scott J. R. Barnett T. C. (2006). Surface proteins of gram-positive bacteria and how they get there. Annu. Rev. Microbiol. 60, 397–423. 10.1146/annurev.micro.60.080805.142256 16753030

[B41] Tonthat H. Liu G. Mazmanian S. K. Faull K. F. Schneewind O. (1999). Purification and characterization of sortase, the transpeptidase that cleaves surface proteins of *Staphylococcus aureus* at the LPXTG motif. Proc. Natl. Acad. Sci. U.S.A. 96:12424 10.1073/pnas.96.22.12424 10535938 PMC22937

[B42] Wallockrichards D. J. Marleswright J. Clarke D. J. Maitra A. Dodds M. Hanley B. (2015). Molecular basis of *Streptococcus mutans* sortase A inhibition by the flavonoid natural product trans-chalcone. Chem. Commun. 51:10483 10.1039/C5CC01816A 26029850

[B43] Wardenburg J. B. Patel R. J. Schneewind O. (2007). Surface proteins and exotoxins are required for the pathogenesis of *Staphylococcus aureus* pneumonia. Infect. Immun. 75, 1040–1044. 10.1128/IAI.01313-06 17101657 PMC1828520

[B44] Zhang B. Wang X. Wang L. Chen S. Shi D. Wang H. (2016). Molecular mechanism of the flavonoid natural product dryocrassin ABBA against *Staphylococcus aureus* sortase A. Molecules 21:1428. 10.3390/molecules21111428 27792196 PMC6273746

[B45] Zhang J. Liu H. Zhu K. Gong S. Dramsi S. Wang Y. T.. (2014). Antiinfective therapy with a small molecule inhibitor of *Staphylococcus aureus* sortase. Proc. Natl. Acad. Sci. U.S.A. 111, 13517–13522. 10.1073/pnas.1408601111 25197057 PMC4169930

[B46] Zhou X. Liu S. Li W. Zhang B. Liu B. Liu Y.. (2015). Phloretin derived from apple can reduce alpha-hemolysin expression in methicillin-resistant *Staphylococcus aureus* USA300. World J. Microbiol. Biotechnol. 31, 1259–1265. 10.1007/s11274-015-1879-1 26026280

